# The complete mitochondrial genome of *Sanghuangporus sanghuang* (Hymenochaetaceae, Basidiomycota)

**DOI:** 10.1080/23802359.2018.1462116

**Published:** 2018-04-12

**Authors:** Jae-Gu Han, Junsang Oh, Jong Won Jo, Chang Sun Kim, Young-Nam Kwag, Sang-Kuk Han, Gi-Ho Sung

**Affiliations:** a Mushroom Research Division, National Institute of Horticultural and Herbal Science, Rural Development Administration, Eumseong, Republic of Korea;; b College of Pharmacy, Chung-Ang University, Seoul, Republic of Korea;; c Forest Biodiversity Division, Korea National Arboretum, Pocheon, Republic of Korea;; d International St. Mary’s Hospital and College of Medicine, Catholic Kwandong University, Incheon, Republic of Korea

**Keywords:** *Sanghuangporus sanghuang*, mushroom, mitochondrial genome, Hymenochaetaceae

## Abstract

Sanghuang is a polypore mushroom, which has been widely used in oriental medicine. Since recent molecular phylogenetic studies elucidated its species delimitation, *Sanghaungporus sanghuang* became the official name of this fungus. In this study, the complete sequence of the mitochondrial DNA of *S. sanghuang* was determined. The whole genome was 112,060 bp containing 14 proteins, 2 ribosomal RNA subunits, and 45 transfer RNAs. The overall GC content of the genome was 23.21%. A neighbour-joining tree based on atp6 sequence data showed its close relationship with the species of *Ganoderma* and *Trametes*.

Sanghuang is a polypore belonging to Hymenochaetaceae in Basidiomycota. Its medicinal benefits such as enhancing detoxication and immune boosting have been intensively studied for the past decades (Chen et al. 2006; Guo et al. 2007; Zhu et al. 2008). The term sanghuang has been widely applied to *Phellinus linteus* (Berk and M.A. Curtis) Teng and its allies in Asian countries (Wu et al. 2012). However, detailed morphological (Dai and Xu 1998; Lim et al. 2003; Dai 2010) and molecular phylogenetic studies (Wagner and Fischer 2001; Larsson et al. 2006; Wu et al. 2012; Zhou et al. 2016) showed that the correct name is not *P. linteus*, but *Sanghuangporus sanghuang* (Sheng H. Wu, T. Hatt. and Y.C. Dai).

The purpose of this study is to characterize the complete mitochondrial genome of *S. sanghuang*. It will contribute to construct a more comprehensive and natural taxonomic system of this group, as well as to design useful molecular markers to discriminate closely related taxa, which are morphologically similar.

KACC54185 was obtained from the Korean Agricultural Culture Collection (KACC), Republic of Korea. Its mycelium was harvested after 2 weeks incubation on malt extract agar (Difco Laboratories, Detroit, MI, USA). Genomic DNA was extracted using the methodology of Lee and Taylor (1990). Next-generation sequencing was performed using PacBio-RS-II and Hiseq2500 following the manufacturers’ instructions. At first, hierarchical genome assembly process (HGAP) was conducted with the PacBio reads. After mapping the Hiseq reads, de novo assembly of the mitochondrial genomes was completed. GeSeq (Annotation of Organellar Genomes) program (Tillich et al. 2017) predicted the mitochondrial gene regions, which were double-checked using blast search and Artemis (Rutherford et al. 2000). The complete and annotated mitochondrial genome has been deposited to GenBank with the accession number of MG149786.

The mitochondrial genome of *S. sanghuang* was represented by linear DNA molecules terminating with inverted repeats (IRs), which may play a role of telomere (Kolesnikov and Gerasimov 2012). Linear mitochondrial genomes induced by IRs have been reported in some fungal species (Forget et al. 2002; Salavirta et al. 2014). The total length was 112,060 bp, which is the second largest among the hitherto reported mitogenomes of polypores (Wang et al. 2016). It contains 2 ribosomal RNA subunits (*rns* and *rnl*), 45 transfer RNA genes, and 14 protein-coding genes of an apocytochrome b (*cob*), three cytochrome c oxidases (*cox1*, *cox2*, and *cox3*), three ATPases (*atp6*, *atp8*, and *atp9*), and seven NADH dehydrogenases (*nad1*, *nad2*, *nad3*, *nad4*, *nad4L*, *nad5*, and *nad6*). The base composition was 38.37% A, 38.41% T, 12.14%G, and 11.07%C with an AT bias of 76.78%.

To ascertain the position of *S. sanghuang* within the Agaricomycotina, a neighbour-joining tree was constructed with MEGA6 (Tamura et al. 2013) using a*tp6* of representative species belonging to Agaricomycotina. The inferred tree represented that sanghuang (Hymenochaetaceae) forms a sister clade of *Ganoderma lucidum, G. sinense* (Ganodermataceae), and *Trametes cingulata* (Polyporaceae) (Figure 1). This is consistent with the traditional classification of the Agaricomycotina with robust bootstrap supports.

**Figure 1.**
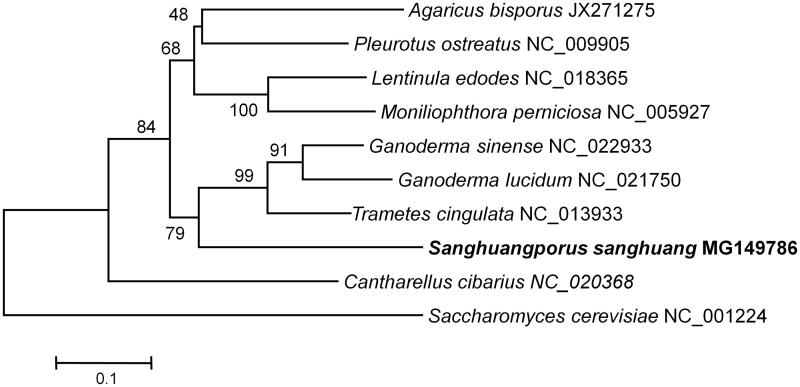
The phylogenetic position of *S. sangahuang* among the selected species of Agaricomycotina. The tree was inferred from neighbour-joining method based on *atp6* nucleotide sequences. Bootstrap values greater than 50% are shown above the corresponding nodes.
